# Bis(2,4,6-triamino-1,3,5-triazin-1-ium) hydrogen phosphate trihydrate

**DOI:** 10.1107/S1600536809054798

**Published:** 2009-12-24

**Authors:** Xue-Mei Li, Si-Si Feng, Fan Wang, Qi Ma, Miao-Li Zhu

**Affiliations:** a College of Chemistry and Chemical Engineering, Shanxi Datong University, Datong, Shanxi 037009, People’s Republic of China; b Institute of Molecular Science, Key Laboratory of Chemical Biology and Molecular, Engineering of the Education Ministry, Shanxi University, Taiyuan, Shanxi 030006, People’s Republic of China

## Abstract

In the title hydrated mol­ecular salt, 2C_3_H_7_N_6_^+^·HPO_4_^2−^·3H_2_O, three of the O atoms of the hydrogen phosphate anion are disordered over two positions, with relative occupancies of 0.763 (1) and 0.237 (1). In the crystal, the components are linked by N—H⋯N, N—H⋯O and O—H⋯O hydrogen bonds

## Related literature

For related structures, see: Choi *et al.* (2004 ▶); Janczak & Perpétuo (2001*a* ▶,*b* ▶,*c* ▶, 2002 ▶, 2003 ▶, 2004 ▶); Li *et al.* (2005 ▶); Perpétuo & Janczak (2002 ▶); Zhang *et al.* (2004 ▶).
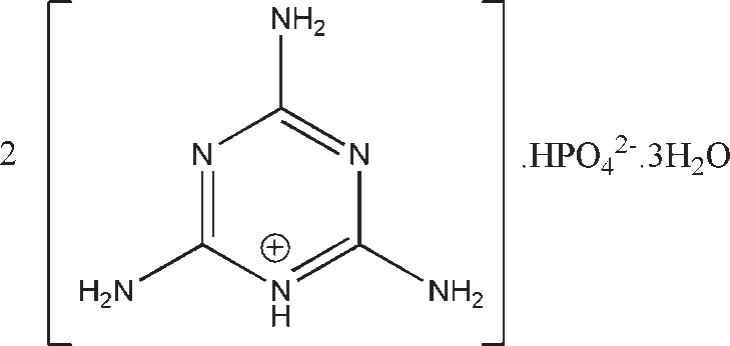


## Experimental

### 

#### Crystal data

2C_3_H_7_N_6_^+^·HPO_4_^2−^·3H_2_O
*M* *_r_* = 404.32
Triclinic, 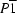

*a* = 6.767 (4) Å
*b* = 10.548 (7) Å
*c* = 12.497 (8) Å
α = 91.865 (11)°
β = 105.609 (10)°
γ = 108.020 (9)°
*V* = 810.3 (9) Å^3^
*Z* = 2
Mo *K*α radiation
μ = 0.24 mm^−1^
*T* = 296 K
0.30 × 0.25 × 0.20 mm

#### Data collection

Bruker SMART 1K CCD diffractometer
Absorption correction: multi-scan (*SADABS*; Bruker, 2000 ▶) *T*_min_ = 0.933, *T*_max_ = 0.954
3823 measured reflections
2803 independent reflections
2027 reflections with *I* > 2σ(*I*)
*R*_int_ = 0.029

#### Refinement

*R*[*F* ^2^ > 2σ(*F* ^2^)] = 0.057
*wR*(*F* ^2^) = 0.178
*S* = 1.06
2803 reflections
274 parameters
H atoms treated by a mixture of independent and constrained refinement
Δρ_max_ = 0.33 e Å^−3^
Δρ_min_ = −0.53 e Å^−3^

### 

Data collection: *SMART* (Bruker, 2000 ▶); cell refinement: *SAINT* (Bruker, 2000 ▶); data reduction: *SAINT*; program(s) used to solve structure: *SHELXS97* (Sheldrick, 2008 ▶); program(s) used to refine structure: *SHELXL97* (Sheldrick, 2008 ▶); molecular graphics: *ORTEP-3* (Farrugia, 1997 ▶); software used to prepare material for publication: *SHELXTL* (Sheldrick, 2008 ▶).

## Supplementary Material

Crystal structure: contains datablocks I, global. DOI: 10.1107/S1600536809054798/hb5248sup1.cif

Structure factors: contains datablocks I. DOI: 10.1107/S1600536809054798/hb5248Isup2.hkl

Additional supplementary materials:  crystallographic information; 3D view; checkCIF report

## Figures and Tables

**Table 1:** Hydrogen-bond geometry (Å, °)

*D*—H⋯*A*	*D*—H	H⋯*A*	*D*⋯*A*	*D*—H⋯*A*
N3—H3⋯O5^i^	0.77 (4)	2.06 (4)	2.791 (4)	158 (4)
N4—H4*B*⋯O4*A*^ii^	0.86	2.22	2.998 (5)	151
N4—H4*A*⋯N7^iii^	0.86	2.20	3.059 (5)	177
N5—H5*B*⋯O7	0.86	1.97	2.822 (5)	170
N5—H5*A*⋯O6^iv^	0.86	2.41	3.258 (5)	167
N6—H6*B*⋯O5^i^	0.86	2.28	3.009 (5)	143
N6—H6*A*⋯N9^v^	0.86	2.17	3.031 (5)	176
N8—H8⋯O3*A*	0.84 (3)	1.93 (4)	2.742 (7)	164 (3)
N10—H10*B*⋯O2*A*	0.86	2.11	2.952 (5)	168
N10—H10*A*⋯O2*A*^vi^	0.86	1.99	2.744 (5)	146
N11—H11*B*⋯O4*A*^vii^	0.86	2.53	3.147 (6)	129
N11—H11*B*⋯O1^vii^	0.86	2.33	3.121 (5)	152
N11—H11*A*⋯N1^v^	0.86	2.02	2.884 (4)	178
N12—H12*B*⋯O6^viii^	0.86	2.07	2.840 (5)	149
N12—H12*A*⋯N2^iii^	0.86	2.08	2.935 (4)	172
O1—H1⋯O3*A*^vii^	0.82	1.76	2.535 (5)	157
O5—H5*D*⋯O3*A*^i^	0.85	1.89	2.739 (5)	177
O5—H5*C*⋯O4*A*	0.85	2.00	2.726 (6)	142
O6—H6*D*⋯O1^ix^	0.85	1.97	2.787 (4)	161
O6—H6*C*⋯O4*A*	0.85	2.02	2.838 (6)	162
O7—H7*B*⋯O1^ix^	0.85	2.57	3.257 (5)	139
O7—H7*B*⋯O2*A*^ix^	0.85	2.28	2.958 (6)	137
O7—H7*A*⋯O5^i^	0.85	2.28	3.006 (6)	144

Symmetry codes: (i) 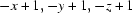; (ii) 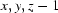; (iii) 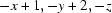; (iv) 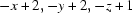; (v) 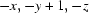; (vi) 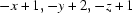; (vii) 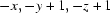; (viii) 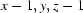; (ix) 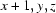.

**Fig. 1.**
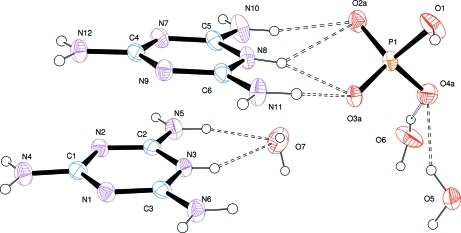
The structure of (I), with displacement ellipsoids drawn at the 30% probability level for non-H atoms.

**Fig. 2.**
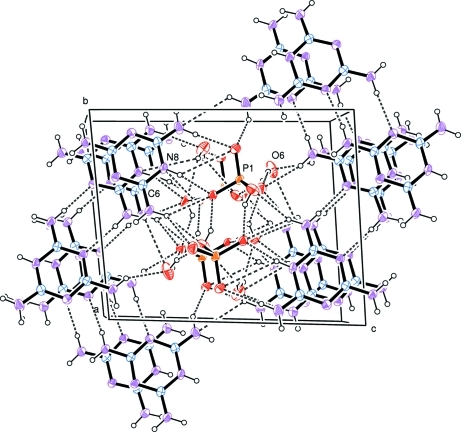
The packing of (I) viewed down the a axis.

## supplementary crystallographic information

### Comment

In previous work, we reported a compound of 2,4,6-triamino-1,3,5-triazin-1-ium with organic anion linked *via* weak interactions (Li *et al.*, 2005). As continuing interest, here, we present the results of the crystal structure of another ion pair adduct containing monoprotonated melaminium, inorganic anion (hydrogen phosphate), and solvent water, (I, Fig. 1 & Table 1).

The aromatic rings both monoprotonated melaminium exhibit significant distortions from the ideal hexagonal form. Thus, the internal C—N—C angles at the protonated N atoms (C1—N3—C2 and C6 N8 C5) are significantly greater than the other two ring (*i.e.* C1—N2—C2, C1—N1—C3, C5—N7—C4, C6—N9—C4) angles, and the internal N2—C1—N1and N7—C4—N9 angle, *i.e.* containing only non-protonated N atoms is greater than either of the remaining N—C—N angles containing both protonated and non-protonated N atoms (Table 1). This feature of the structure is similar to our previous report (Li *et al.*, 2005) and the other reported mono-protonated melaminium cations (Janczak & Perpétuo, 2001*a*,b,c, 2002, 2003, 2004; Perpétuo & Janczak, 2002; Zhang *et al.*, 2004; Choi *et al.*, 2004).

Between charged residues and the water molecules interact extensively by a combination of ionic, H-bonds (Table 2) as well as π-π interactions as shown in Fig 2. Neighboring melaminium residues are interconnected by double N—H···N H-bonds, leading to the formation of layers.

### Experimental

Hot solutions of melamine and *meta*-phosphoric acid in a 1:1 molar ratio were mixed and, after allowed the mixture to stand at room temperature for a few days, colourless blocks of (I) were desposited.

### Refinement

H atoms attached to N (except N3 and N8) and O1 atoms of (I) were placed in geometrically idealized positions with N—H = 0.86 Å O1—H = 0.82 Å and refined with *U*_iso_=1.2*U*_eq_ [N] or *U*_iso_=1.5*U*_eq_ [O1]. H atoms attached to O (water) N3 and N8 atom were located from the difference Fourier maps and refined their global *U*_iso_=1.2*U*_eq_. The N3—H, N8—H, and O—H distances are in the range 0.768 - 0.851 Å. The O2, O3, and, O4 atoms from hydrogen phosphate anion of crystallization in (I) were found to be disordered and were modelled over two sets of positions using restraints on the anisotropic displacement parameters of these O atoms. The major and minor disorder components had refined occupancies of O2A 0.77 (1), O3A 0.76 (1), O4A 0.76 (1) % and O2B 0.23 (1) O3B 0.24 (1)O4B 0.24 (1)%, respectively.

### Crystal data

**Table:**

2C_3_H_7_N_6_^+^·HPO_4_^2^^−^·3H_2_O	*Z* = 2
*M**_r_* = 404.32	*F*(000) = 424
Triclinic, *P*1	*D*_x_ = 1.657 Mg m^−^^3^
Hall symbol: -P 1	Mo *K*α radiation, λ = 0.71073 Å
*a* = 6.767 (4) Å	Cell parameters from 1970 reflections
*b* = 10.548 (7) Å	θ = 2.5–28.0°
*c* = 12.497 (8) Å	µ = 0.24 mm^−^^1^
α = 91.865 (11)°	*T* = 296 K
β = 105.609 (10)°	Block, colourless
γ = 108.020 (9)°	0.30 × 0.25 × 0.20 mm
*V* = 810.3 (9) Å^3^	

### Data collection

**Table:**

Bruker SMART 1K CCD diffractometer	2803 independent reflections
Radiation source: fine-focus sealed tube	2027 reflections with *I* > 2σ(*I*)
graphite	*R*_int_ = 0.029
ω scans	θ_max_ = 25.1°, θ_min_ = 2.1°
Absorption correction: multi-scan (*SADABS*; Bruker, 2000)	*h* = −7→8
*T*_min_ = 0.933, *T*_max_ = 0.954	*k* = −11→12
3823 measured reflections	*l* = −14→7

### Refinement

**Table:**

Refinement on *F*^2^	Primary atom site location: structure-invariant direct methods
Least-squares matrix: full	Secondary atom site location: difference Fourier map
*R*[*F*^2^ > 2σ(*F*^2^)] = 0.057	Hydrogen site location: inferred from neighbouring sites
*wR*(*F*^2^) = 0.178	H atoms treated by a mixture of independent and constrained refinement
*S* = 1.06	*w* = 1/[σ^2^(*F*_o_^2^) + (0.0996*P*)^2^ + 0.520*P*] where *P* = (*F*_o_^2^ + 2*F*_c_^2^)/3
2803 reflections	(Δ/σ)_max_ = 0.003
274 parameters	Δρ_max_ = 0.33 e Å^−^^3^
0 restraints	Δρ_min_ = −0.53 e Å^−^^3^

### Special details

**Table:**

Geometry. All e.s.d.'s (except the e.s.d. in the dihedral angle between two l.s. planes) are estimated using the full covariance matrix. The cell e.s.d.'s are taken into account individually in the estimation of e.s.d.'s in distances, angles and torsion angles; correlations between e.s.d.'s in cell parameters are only used when they are defined by crystal symmetry. An approximate (isotropic) treatment of cell e.s.d.'s is used for estimating e.s.d.'s involving l.s. planes.
Refinement. Refinement of *F*^2^ against ALL reflections. The weighted *R*-factor *wR* and goodness of fit *S* are based on *F*^2^, conventional *R*-factors *R* are based on *F*, with *F* set to zero for negative *F*^2^. The threshold expression of *F*^2^ > σ(*F*^2^) is used only for calculating *R*-factors(gt) *etc*. and is not relevant to the choice of reflections for refinement. *R*-factors based on *F*^2^ are statistically about twice as large as those based on *F*, and *R*- factors based on ALL data will be even larger.

### Fractional atomic coordinates and isotropic or equivalent isotropic displacement parameters (Å^2^)

**Table:**

	*x*	*y*	*z*	*U*_iso_*/*U*_eq_	Occ. (<1)
C1	0.5323 (6)	0.7920 (4)	−0.0156 (3)	0.0352 (8)	
C2	0.6740 (6)	0.8584 (4)	0.1714 (3)	0.0359 (8)	
C3	0.4373 (6)	0.6393 (4)	0.1008 (3)	0.0337 (8)	
C4	0.0847 (5)	0.8128 (3)	0.0751 (3)	0.0317 (8)	
C5	0.2296 (6)	0.8597 (4)	0.2627 (3)	0.0372 (8)	
C6	−0.0212 (5)	0.6489 (4)	0.1803 (3)	0.0329 (8)	
N1	0.4191 (5)	0.6664 (3)	−0.0031 (2)	0.0352 (7)	
N2	0.6575 (5)	0.8903 (3)	0.0688 (2)	0.0355 (7)	
N3	0.5651 (5)	0.7323 (3)	0.1889 (3)	0.0363 (7)	
H3	0.577 (6)	0.712 (4)	0.248 (3)	0.029 (10)*	
N4	0.5233 (5)	0.8212 (3)	−0.1176 (3)	0.0436 (8)	
H4A	0.5955	0.9002	−0.1280	0.052*	
H4B	0.4453	0.7613	−0.1741	0.052*	
N5	0.7957 (5)	0.9458 (3)	0.2586 (3)	0.0459 (8)	
H5A	0.8672	1.0258	0.2499	0.055*	
H5B	0.8047	0.9235	0.3249	0.055*	
N6	0.3325 (5)	0.5192 (3)	0.1207 (3)	0.0418 (8)	
H6A	0.2515	0.4577	0.0659	0.050*	
H6B	0.3450	0.5021	0.1886	0.050*	
N7	0.2203 (5)	0.9023 (3)	0.1634 (2)	0.0358 (7)	
N8	0.1115 (5)	0.7333 (3)	0.2726 (3)	0.0374 (7)	
H8	0.126 (5)	0.703 (3)	0.334 (3)	0.021 (8)*	
N9	−0.0400 (4)	0.6864 (3)	0.0791 (2)	0.0330 (7)	
N10	0.3541 (5)	0.9350 (4)	0.3562 (3)	0.0552 (10)	
H10A	0.4343	1.0157	0.3550	0.066*	
H10B	0.3558	0.9039	0.4191	0.066*	
N11	−0.1322 (5)	0.5270 (3)	0.1931 (3)	0.0420 (8)	
H11A	−0.2178	0.4709	0.1354	0.050*	
H11B	−0.1193	0.5031	0.2591	0.050*	
N12	0.0741 (5)	0.8512 (3)	−0.0246 (2)	0.0379 (7)	
H12A	0.1520	0.9305	−0.0310	0.045*	
H12B	−0.0107	0.7969	−0.0834	0.045*	
P1	0.24796 (15)	0.68587 (9)	0.57240 (7)	0.0335 (3)	
O1	0.0294 (5)	0.6330 (3)	0.6053 (3)	0.0599 (9)	
H1	−0.0482	0.5614	0.5669	0.090*	
O2A	0.2963 (6)	0.8336 (3)	0.5671 (3)	0.0445 (14)	0.767 (9)
O3A	0.2088 (7)	0.6098 (4)	0.4601 (4)	0.0353 (15)	0.757 (14)
O4A	0.4058 (6)	0.6486 (5)	0.6643 (3)	0.0507 (16)	0.765 (11)
O2B	0.176 (2)	0.7686 (13)	0.4856 (10)	0.046 (5)	0.233 (9)
O3B	0.279 (2)	0.5614 (14)	0.5130 (13)	0.040 (5)	0.243 (14)
O4B	0.4481 (19)	0.7567 (17)	0.6642 (10)	0.048 (5)	0.235 (11)
O5	0.4731 (5)	0.4107 (3)	0.6295 (3)	0.0606 (9)	
H5C	0.4405	0.4792	0.6078	0.091*	
H5D	0.5757	0.4063	0.6041	0.091*	
O6	0.8660 (5)	0.7583 (4)	0.7442 (3)	0.0753 (11)	
H6C	0.7299	0.7431	0.7189	0.113*	
H6D	0.8984	0.7038	0.7061	0.113*	
O7	0.8612 (6)	0.8583 (4)	0.4724 (3)	0.0799 (11)	
H7A	0.7320	0.8059	0.4420	0.120*	
H7B	0.9418	0.8093	0.4809	0.120*	

### Atomic displacement parameters (Å^2^)

**Table:**

	*U*^11^	*U*^22^	*U*^33^	*U*^12^	*U*^13^	*U*^23^
C1	0.0292 (18)	0.040 (2)	0.040 (2)	0.0123 (16)	0.0150 (15)	0.0077 (16)
C2	0.0342 (19)	0.039 (2)	0.042 (2)	0.0175 (16)	0.0177 (16)	0.0081 (17)
C3	0.0292 (18)	0.038 (2)	0.040 (2)	0.0133 (15)	0.0165 (15)	0.0067 (16)
C4	0.0274 (18)	0.039 (2)	0.0343 (19)	0.0133 (15)	0.0146 (15)	0.0090 (15)
C5	0.0281 (18)	0.047 (2)	0.035 (2)	0.0074 (16)	0.0143 (15)	0.0034 (16)
C6	0.0272 (18)	0.044 (2)	0.0321 (19)	0.0140 (16)	0.0127 (14)	0.0088 (15)
N1	0.0342 (17)	0.0360 (17)	0.0369 (17)	0.0086 (13)	0.0161 (13)	0.0076 (13)
N2	0.0348 (16)	0.0349 (17)	0.0402 (17)	0.0088 (13)	0.0193 (13)	0.0082 (13)
N3	0.0397 (18)	0.0396 (18)	0.0358 (19)	0.0147 (14)	0.0188 (15)	0.0119 (15)
N4	0.0468 (19)	0.0413 (18)	0.0375 (18)	0.0038 (15)	0.0161 (14)	0.0071 (14)
N5	0.054 (2)	0.0432 (19)	0.0371 (18)	0.0088 (15)	0.0154 (15)	0.0043 (15)
N6	0.0464 (19)	0.0408 (19)	0.0396 (18)	0.0101 (15)	0.0188 (15)	0.0120 (14)
N7	0.0302 (16)	0.0381 (17)	0.0375 (17)	0.0053 (13)	0.0146 (13)	0.0040 (13)
N8	0.0384 (18)	0.047 (2)	0.0266 (17)	0.0108 (15)	0.0130 (14)	0.0108 (14)
N9	0.0303 (15)	0.0344 (17)	0.0331 (16)	0.0065 (13)	0.0119 (12)	0.0084 (12)
N10	0.048 (2)	0.062 (2)	0.0367 (19)	−0.0071 (17)	0.0125 (16)	−0.0012 (16)
N11	0.0438 (18)	0.0413 (18)	0.0323 (17)	0.0017 (14)	0.0111 (14)	0.0125 (13)
N12	0.0408 (18)	0.0368 (17)	0.0337 (17)	0.0056 (14)	0.0150 (13)	0.0101 (13)
P1	0.0332 (5)	0.0360 (5)	0.0311 (5)	0.0064 (4)	0.0142 (4)	0.0090 (4)
O1	0.0576 (19)	0.0512 (19)	0.068 (2)	−0.0043 (14)	0.0431 (16)	−0.0138 (15)
O2A	0.047 (2)	0.039 (2)	0.047 (3)	0.0066 (17)	0.022 (2)	0.0108 (18)
O3A	0.036 (2)	0.037 (2)	0.033 (2)	0.0065 (16)	0.0155 (18)	0.0097 (17)
O4A	0.058 (3)	0.059 (4)	0.038 (2)	0.028 (2)	0.0091 (17)	0.0085 (18)
O2B	0.060 (9)	0.058 (9)	0.030 (8)	0.032 (7)	0.015 (7)	0.015 (6)
O3B	0.042 (7)	0.045 (8)	0.039 (9)	0.019 (6)	0.015 (6)	−0.006 (6)
O4B	0.039 (7)	0.054 (12)	0.047 (7)	0.012 (6)	0.008 (5)	0.011 (6)
O5	0.073 (2)	0.075 (2)	0.065 (2)	0.0440 (18)	0.0458 (17)	0.0388 (16)
O6	0.065 (2)	0.121 (3)	0.0487 (19)	0.050 (2)	0.0113 (16)	−0.0150 (18)
O7	0.085 (3)	0.086 (3)	0.077 (2)	0.036 (2)	0.025 (2)	0.037 (2)

### Geometric parameters (Å, °)

**Table:**

C1—N4	1.311 (5)	N6—H6A	0.8600
C1—N2	1.346 (5)	N6—H6B	0.8600
C1—N1	1.347 (5)	N8—H8	0.84 (3)
C2—N5	1.303 (5)	N10—H10A	0.8600
C2—N2	1.320 (5)	N10—H10B	0.8600
C2—N3	1.363 (5)	N11—H11A	0.8600
C3—N6	1.315 (5)	N11—H11B	0.8600
C3—N1	1.320 (4)	N12—H12A	0.8600
C3—N3	1.347 (5)	N12—H12B	0.8600
C4—N12	1.313 (4)	P1—O4B	1.480 (12)
C4—N7	1.343 (5)	P1—O2B	1.488 (10)
C4—N9	1.350 (4)	P1—O2A	1.498 (4)
C5—N10	1.305 (5)	P1—O4A	1.498 (4)
C5—N7	1.326 (5)	P1—O3A	1.510 (4)
C5—N8	1.355 (5)	P1—O1	1.581 (3)
C6—N11	1.313 (5)	P1—O3B	1.584 (11)
C6—N9	1.323 (4)	O1—H1	0.8200
C6—N8	1.346 (5)	O5—H5C	0.8501
N3—H3	0.77 (4)	O5—H5D	0.8500
N4—H4A	0.8600	O6—H6C	0.8500
N4—H4B	0.8600	O6—H6D	0.8501
N5—H5A	0.8600	O7—H7A	0.8508
N5—H5B	0.8600	O7—H7B	0.8500
			
N4—C1—N2	116.9 (3)	H6A—N6—H6B	120.0
N4—C1—N1	118.0 (3)	C5—N7—C4	115.4 (3)
N2—C1—N1	125.1 (3)	C6—N8—C5	119.8 (3)
N5—C2—N2	121.1 (3)	C6—N8—H8	118 (2)
N5—C2—N3	118.1 (3)	C5—N8—H8	122 (2)
N2—C2—N3	120.7 (3)	C6—N9—C4	115.6 (3)
N6—C3—N1	120.4 (3)	C5—N10—H10A	120.0
N6—C3—N3	118.3 (3)	C5—N10—H10B	120.0
N1—C3—N3	121.3 (3)	H10A—N10—H10B	120.0
N12—C4—N7	117.0 (3)	C6—N11—H11A	120.0
N12—C4—N9	116.9 (3)	C6—N11—H11B	120.0
N7—C4—N9	126.1 (3)	H11A—N11—H11B	120.0
N10—C5—N7	122.6 (4)	C4—N12—H12A	120.0
N10—C5—N8	115.9 (3)	C4—N12—H12B	120.0
N7—C5—N8	121.5 (3)	H12A—N12—H12B	120.0
N11—C6—N9	120.4 (3)	O4B—P1—O2B	116.0 (8)
N11—C6—N8	118.1 (3)	O2A—P1—O4A	115.2 (3)
N9—C6—N8	121.5 (3)	O2A—P1—O3A	112.0 (2)
C3—N1—C1	116.4 (3)	O4A—P1—O3A	112.0 (3)
C2—N2—C1	116.6 (3)	O4B—P1—O1	116.8 (5)
C3—N3—C2	119.9 (3)	O2B—P1—O1	97.5 (5)
C3—N3—H3	118 (3)	O2A—P1—O1	107.06 (18)
C2—N3—H3	122 (3)	O4A—P1—O1	102.7 (2)
C1—N4—H4A	120.0	O3A—P1—O1	106.93 (17)
C1—N4—H4B	120.0	O4B—P1—O3B	109.3 (8)
H4A—N4—H4B	120.0	O2B—P1—O3B	108.8 (9)
C2—N5—H5A	120.0	O1—P1—O3B	107.7 (4)
C2—N5—H5B	120.0	P1—O1—H1	109.5
H5A—N5—H5B	120.0	H5C—O5—H5D	107.7
C3—N6—H6A	120.0	H6C—O6—H6D	107.7
C3—N6—H6B	120.0	H7A—O7—H7B	106.2
			
N6—C3—N1—C1	−179.9 (3)	N10—C5—N7—C4	179.9 (4)
N3—C3—N1—C1	−0.9 (5)	N8—C5—N7—C4	1.0 (5)
N4—C1—N1—C3	177.9 (3)	N12—C4—N7—C5	−179.0 (3)
N2—C1—N1—C3	−1.1 (5)	N9—C4—N7—C5	0.1 (5)
N5—C2—N2—C1	178.3 (3)	N11—C6—N8—C5	179.6 (3)
N3—C2—N2—C1	−1.1 (5)	N9—C6—N8—C5	−0.2 (5)
N4—C1—N2—C2	−176.9 (3)	N10—C5—N8—C6	−180.0 (3)
N1—C1—N2—C2	2.1 (5)	N7—C5—N8—C6	−1.0 (5)
N6—C3—N3—C2	−179.2 (3)	N11—C6—N9—C4	−178.6 (3)
N1—C3—N3—C2	1.8 (5)	N8—C6—N9—C4	1.2 (5)
N5—C2—N3—C3	179.9 (3)	N12—C4—N9—C6	178.0 (3)
N2—C2—N3—C3	−0.8 (5)	N7—C4—N9—C6	−1.2 (5)

### Hydrogen-bond geometry (Å, °)

**Table:**

*D*—H···*A*	*D*—H	H···*A*	*D*···*A*	*D*—H···*A*
N3—H3···O5^i^	0.77 (4)	2.06 (4)	2.791 (4)	158 (4)
N4—H4B···O4A^ii^	0.86	2.22	2.998 (5)	151
N4—H4A···N7^iii^	0.86	2.20	3.059 (5)	177
N5—H5B···O7	0.86	1.97	2.822 (5)	170
N5—H5A···O6^iv^	0.86	2.41	3.258 (5)	167
N6—H6B···O5^i^	0.86	2.28	3.009 (5)	143
N6—H6A···N9^v^	0.86	2.17	3.031 (5)	176
N8—H8···O3A	0.84 (3)	1.93 (4)	2.742 (7)	164 (3)
N10—H10B···O2A	0.86	2.11	2.952 (5)	168
N10—H10A···O2A^vi^	0.86	1.99	2.744 (5)	146
N11—H11B···O4A^vii^	0.86	2.53	3.147 (6)	129
N11—H11B···O1^vii^	0.86	2.33	3.121 (5)	152
N11—H11A···N1^v^	0.86	2.02	2.884 (4)	178
N12—H12B···O6^viii^	0.86	2.07	2.840 (5)	149
N12—H12A···N2^iii^	0.86	2.08	2.935 (4)	172
O1—H1···O3A^vii^	0.82	1.76	2.535 (5)	157
O5—H5D···O3A^i^	0.85	1.89	2.739 (5)	177
O5—H5C···O4A	0.85	2.00	2.726 (6)	142
O6—H6D···O1^ix^	0.85	1.97	2.787 (4)	161
O6—H6C···O4A	0.85	2.02	2.838 (6)	162
O7—H7B···O1^ix^	0.85	2.57	3.257 (5)	139
O7—H7B···O2A^ix^	0.85	2.28	2.958 (6)	137
O7—H7A···O5^i^	0.85	2.28	3.006 (6)	144

Symmetry codes: (i) −*x*+1, −*y*+1, −*z*+1; (ii) *x*, *y*, *z*−1; (iii) −*x*+1, −*y*+2, −*z*; (iv) −*x*+2, −*y*+2, −*z*+1; (v) −*x*, −*y*+1, −*z*; (vi) −*x*+1, −*y*+2, −*z*+1; (vii) −*x*, −*y*+1, −*z*+1; (viii) *x*−1, *y*, *z*−1; (ix) *x*+1, *y*, *z*.
